# An evaluation of the effects of lowering blood alcohol concentration limits for drivers on the rates of road traffic accidents and alcohol consumption: a natural experiment

**DOI:** 10.1016/S0140-6736(18)32850-2

**Published:** 2019-01-26

**Authors:** Houra Haghpanahan, Jim Lewsey, Daniel F Mackay, Emma McIntosh, Jill Pell, Andy Jones, Niamh Fitzgerald, Mark Robinson

**Affiliations:** a Health Economics and Health Technology Assessment, University of Glasgow, Glasgow, UK; b Public Health, University of Glasgow, Glasgow, UK; c Institute of Health and Wellbeing, University of Glasgow, Glasgow, UK; d Norwich Medical School, University of East Anglia, Norwich, UK; e Institute for Social Marketing, UK Centre for Tobacco & Alcohol Studies, Faculty of Health Sciences & Sport, University of Stirling, Stirling, UK; f Public Health Observatory, NHS Health Scotland, Glasgow, UK

## Abstract

**Background:**

Drink driving is an important risk factor for road traffic accidents (RTAs), which cause high levels of morbidity and mortality globally. Lowering the permitted blood alcohol concentration (BAC) for drivers is a common public health intervention that is enacted in countries and jurisdictions across the world. In Scotland, on Dec 5, 2014, the BAC limit for drivers was reduced from 0·08 g/dL to 0·05 g/dL. We therefore aimed to evaluate the effects of this change on RTAs and alcohol consumption.

**Methods:**

In this natural experiment, we used an observational, comparative interrupted time-series design by use of data on RTAs and alcohol consumption in Scotland (the interventional group) and England and Wales (the control group). We obtained weekly counts of RTAs from police accident records and we estimated weekly off-trade (eg, in supermarkets and convenience stores) and 4-weekly on-trade (eg, in bars and restaurants) alcohol consumption from market research data. We also used data from automated traffic counters as denominators to calculate RTA rates. We estimated the effect of the intervention on RTAs by use of negative binomial panel regression and on alcohol consumption outcomes by use of seasonal autoregressive integrated moving average models. Our primary outcome was weekly rates of RTAs in Scotland, England, and Wales. This study is registered with ISRCTN, number ISRCTN38602189.

**Findings:**

We assessed the weekly rate of RTAs and alcohol consumption between Jan 1, 2013, and Dec 31, 2016, before and after the BAC limit came into effect on Dec 5, 2014. After the reduction in BAC limits for drivers in Scotland, we found no significant change in weekly RTA rates after adjustment for seasonality and underlying temporal trend (rate ratio 1·01, 95% CI 0·94–1·08; p=0.77) or after adjustment for seasonality, the underlying temporal trend, and the driver characteristics of age, sex, and socioeconomic deprivation (1·00, 0·96–1·06; p=0·73). Relative to RTAs in England and Wales, where the reduction in BAC limit for drivers did not occur, we found a 7% increase in weekly RTA rates in Scotland after this reduction in BAC limit for drivers (1·07, 1·02–1·13; p=0·007 in the fully-adjusted model). Similar findings were observed for serious or fatal RTAs and single-vehicle night-time RTAs. The change in legislation in Scotland was associated with no change in alcohol consumption, measured by per-capita off-trade sales (−0·3%, −1·7 to 1·1; p=0·71), but a 0·7% decrease in alcohol consumption measured by per-capita on-trade sales (−0·7%, −0·8 to −0·5; p<0·0001).

**Interpretation:**

Lowering the driving BAC limit to 0·05 g/dL from 0·08 g/dL in Scotland was not associated with a reduction in RTAs, but this change was associated with a small reduction in per-capita alcohol consumption from on-trade alcohol sales. One plausible explanation is that the legislative change was not suitably enforced—for example with random breath testing measures. Our findings suggest that changing the legal BAC limit for drivers in isolation does not improve RTA outcomes. These findings have significant policy implications internationally as several countries and jurisdictions consider a similar reduction in the BAC limit for drivers.

**Funding:**

National Institute for Health Research Public Health Research Programme.

## Introduction

Road traffic accidents (RTAs) are a major public health problem, with 1·25 million road traffic deaths globally in 2013.^1^ In Great Britain, there have been large reductions in RTAs over recent decades, including a 72% reduction in fatal RTAs observed between 1979 and 2017. However, RTAs remain a considerable burden on health: in 2017, 170 993 casualties from RTAs were reported.^2^ Driving under the influence of alcohol is a major risk factor for RTAs, and a dose-response relation is observed between blood alcohol concentration (BAC) and RTAs. It has been estimated that the odds of fatal injury increase by 1·74 for every 0·02% increase in BAC.^3^ In the UK in 2016, there were at least 6070 RTAs involving a driver with a BAC over the legal limit.^4^

Since Norway introduced a legal BAC limit for driving in 1936, other countries across Europe, North America, Japan, and Australasia have also introduced BAC limits for driving,^5^ initially by introducing a standard BAC limit (of 0·05 g/dL, 0·08 g/dL, or 0·1 g/dL) and with some countries further lowering the limit. In Europe, only England, Wales, and Malta have a 0·08 g/dL BAC limit for drivers. These limits are the norm in many other jurisdictions, including many states in the USA, despite longstanding calls for reductions in the BAC limit for drivers. According to European Commission recommendations,^6^ BAC limits should be set at 0·05 g/dL. The British Road Safety Act introduced a legal limit of 0·08 g/dL in 1967, which is still in effect today. An exception to this law exists in Scotland, where the BAC limit was reduced to 0·05 g/dL on Dec 5, 2014.

**Research in context**

**Evidence before this study**

Road traffic accidents (RTAs) are a major public health problem, with 1·25 million road traffic deaths globally in 2013. There is strong evidence that a person's ability to drive a vehicle is impaired with alcohol in their bloodstream, and drink driving is an important risk factor of RTAs. There is a dose-response relationship between blood alcohol concentration (BAC) and RTA rates, with evidence showing that the odds of fatal injury increase by 1·74 for every 0·02% increase in BAC. There is international evidence that the frequency of severe and fatal RTAs reduce when a country or region changes the legal BAC limit from 0·08 g/dL to 0·05 g/dL. However, these studies have limitations, including confounding of the effect of this BAC intervention by those of other interventions (such as random breath testing) and by poor study design (eg, before-and-after studies that do not account for temporal trends, the absence of a control group, and a low frequency of time-series data). To summarise the highest quality evidence, a European study that analysed data from 15 countries found that an equivalent legislation change was associated with a 7·4% reduction in road fatalities (decreasing to 4·3% after adjustment for random breath testing). An earlier study that evaluated legislation change in two Australian states in the early 1980s found a similar effect size in one state and 14–18% reductions in severe and fatal RTAs in the other. We searched the international scientific literature to identify papers and reports that evaluated either an introduction of BAC limit legislation or evaluated a lowering of a BAC limit. We searched PubMed for related papers published before June 19, 2018, with the search terms “blood alcohol concen*”, “drink AND driving”, “lowering BAC limit”, and “breath test*”, with no language restrictions.

**Added value of this study**

Our findings indicate that the reduction in Scotland's drink-drive limit in December, 2014 did not have the intended effect of reducing RTAs. This reduction in the BAC limit for drivers decreased alcohol consumption from on-trade alcohol sales (eg, in bars and restaurants) by less than 1% but did not affect alcohol consumption from off-trade sales (eg, from supermarkets and convenience stores), which account for approximately three-quarters of total sales. We evaluated the effects of a change in drink-drive legislation from a BAC limit of 0·08 g/dL to 0·05 g/dL in an entire population, which reduced the risk of selection biases. This new evidence is important because the larger effect sizes seen historically might be more difficult to obtain in an era of improved road safety and, regardless of BAC limits, where drink driving is increasingly socially unacceptable.

**Implications of all the available evidence**

This drink-drive limit change occurred in the context of a lack of additional police enforcement and without random breath testing measures in place. Our finding of no effect of the intervention of reducing the BAC driving limit from 0·08 g/dL to 0·05 g/dL supports the hypothesis that enhanced enforcement might be necessary to improve RTA outcomes. However, further research is required to test whether appropriate enforcement of a change in drink-drive legislation from a BAC limit of 0·08 g/dL to 0·05 g/dL would improve outcomes.

The effectiveness of reducing BAC limits has been estimated in countries and jurisdictions within countries that have changed legislation to deter so-called drink driving, which we define as driving with a BAC in excess of the legal limit, to prevent RTAs. Evaluations of the effects of a reduction in driving BAC limits in different parts of the world, such as Australia,^7^ France,^8^ Austria,^9^ and Serbia^10^ provide evidence that such legislation is effective in reducing RTAs. A 2017 meta-analysis^11^ estimated that a standardised reduction in the BAC limit is associated with a 5% decrease in non-fatal alcohol-related traffic accidents, and a reduction to 0·05 g/dL is associated with an 11% decrease in fatal alcohol-related crashes.

In an interrupted time-series study^7^ with a main focus on the evaluation of random breath testing, Henstridge and colleagues assessed the effects of a reduction in BAC limit from 0·08 g/dL to 0·05 g/dL in New South Wales and Queensland, Australia. These changes in BAC limit for drivers were effected in December, 1980 (New South Wales), and December, 1982 (Queensland), and the evaluation was not confounded by random breath testing because this measure was introduced at a later time. The study reported reductions of 7% in the number of severe RTAs and of 8% in the number of fatal RTAs in New South Wales, whereas reductions of 14% in severe RTAs and of 18% in fatal RTAs were reported in Queensland. In a differences-in-differences analysis of data from 15 European countries in 1991–2003, Albalate^12^ found that a BAC limit for drivers of 0·05 g/dL or lower was associated with a 4·5% reduction in road fatality rates with population denominators and with a 7·4% reduction in road fatality rates with per distance driven denominators compared with higher limits. Importantly, these effect sizes reduced to 3·4% (for population denominators) and 4·3% (for per distance driven denominators), and they were no longer statistically significant after adjusting for random breath testing.

If any BAC intervention effect is homogeneous across the population under study then such an intervention could affect absolute inequalities in RTAs that are associated with socioeconomic deprivation: alcohol intake is positively associated with the probability of drink driving,^13^ and greater socioeconomic deprivation is associated with greater alcohol intake per drinker^14^ but also with lower amounts of driving.

It has been hypothesised that alcohol consumption at a population level (ie, per-capita alcohol consumption) is associated with driving under the influence of alcohol.^15^ If correct, an unintended outcome of a change in BAC legislation could be a reduction in per-capita alcohol consumption.

We aimed to evaluate whether lowering the permitted BAC when driving from 0·08 g/dL to 0·05 g/dL in Scotland had an effect on the rate of RTAs and population-level alcohol consumption. Further, we evaluated whether any effects varied by level of socioeconomic deprivation. Our study design allowed us to isolate the effect of changing the legal BAC limit when driving and to assess the sole effect of a change in legislation without any enhanced law enforcement measures such as random breath testing. An appropriate control was provided by neighbouring countries (England and Wales) to the intervention country (Scotland). To our knowledge, no previous study has evaluated whether a legislation change of BAC limit for drivers has led to a reduction in that country's drinking at a population level. These matters are of significant policy importance as other countries and jurisdictions across the world consider similar lowering of BAC limits for drivers.

## Methods

### Study design

In this natural experiment, we used a comparative interrupted time-series design with data before and after the introduction of the lower BAC limit on Dec 5, 2014. The interventional group, where the intervention was introduced, was Scotland, and the control group, where the BAC limit for drivers had not changed, was England and Wales.

### Procedures and outcomes

Our primary outcome was the weekly rates of RTAs in Scotland, England, and Wales. The primary outcome was weekly RTA rates rather than counts, because this measure allows for a differences-in-differences type analysis of effect size. Weekly counts of RTAs that occurred on public roads in the UK and were reported to the police (by use of the STATS19 accident reporting form) were obtained upon request from the Department of Transport. Any given accident could involve more than one driver and casualty, but we considered an accident to be the most appropriate unit for analysis. To calculate weekly RTA rates, the number of miles driven by each person at risk of having an RTA would be the ideal denominator, but these data are not available. As a proxy for this measure, data from automatic traffic counters^16^ which continually count vehicles that pass over them were used. Approximately 300 automatic traffic counters are placed to be representative of the entire UK road network, including motorways, major roads (that provide large-scale transport linkage), and minor roads (that feed traffic between major roads and smaller roads). We accounted for poor quality or missing traffic count data by use of a multiple imputation approach that is specifically designed for time-series data.^17^

In the STATS19 form, the severity of an RTA is recorded as the most severely injured casualty—namely the categories of fatal, serious, or slight injuries. To facilitate comparison with previous studies, and because it can be argued that serious and fatal RTAs are more likely to result from drink driving, we used weekly serious or fatal RTA rates as a secondary outcome. We tested the sensitivity of combining serious with fatal RTAs by modelling each outcome separately. As an additional outcome that was likely to result from drink driving, we used single-vehicle night-time RTAs and the ratio of single-vehicle night-time to multiple vehicle day-time RTAs as secondary outcomes, and we also assessed multi-vehicle day-time RTA outcomes alone.

The final secondary outcome measure, alcohol consumption, was assessed by the volume of pure alcohol sold per-capita in off-trade and on-trade alcohol retail sales. This is a high-quality measure that is not reliant on individual self-reporting, which is prone to bias. These data were provided by NHS Health Scotland for the period 2013–16, who obtained them from the market research company Nielsen.^18^ Off-trade alcohol sales (from retailers licensed to sell alcohol for consumption away from the premises, such as supermarkets and convenience stores) were available in weekly units, but on-trade alcohol sales (from retailers licensed to sell alcohol for consumption on the premises, such as bars and restaurants) were only available in 4-weekly units. We used a linear interpolation method to impute weekly on-trade sales. Per-capita estimates were obtained by dividing the total volume of pure alcohol sold by adult (aged 16 years and older) population size for Scotland, and England and Wales combined.

### Statistical analysis

To assess the comparability of the interventional and control groups, we compared age, sex, and socioeconomic deprivation between people involved in RTAs in these groups. When the RTA involved more than one vehicle, the oldest age group, the most frequent sex, and the highest socioeconomic deprivation level (which was generated from their postcode) of the drivers involved in the accident was used for analysis. We used an area-based measure of socioeconomic deprivation levels separately for Scotland and for England and Wales. Socioeconomic deprivation was measured by use of the Index of Multiple Deprivation that was provided by Scottish and UK Governments.^19^, ^20^, ^21^ As a sensitivity analysis, we repeated our analyses with the opposite rules of demographic assignment (the youngest age group, the least frequent sex, and the lowest socioeconomic deprivation level of the drivers).

To test for a change in RTA counts and rates after the new legislation was in place, we separately fitted negative binomial regression models to panel data sets for the interventional and control groups. To model RTA rates, traffic flows were used as the denominator. We adjusted the models for underlying temporal trend by fitting a covariate representing the week number, and we adjusted for seasonality by use of covariates representing 4-weekly periods of the year (which generated 13 months). The models were then further adjusted for age, sex, and socioeconomic deprivation of the drivers involved in the RTAs. To obtain a differences-in-differences type measure of effect, we appended the two panel data sets, and we assessed an interaction term between interventional group covariate and the binary covariate to indicate the pre-change and post-change in legislation (with a so-called pseudo change for control). In this differences-in-differences type model, an interaction term between week number and the interventional group indicator allowed for a relaxation of the usual differences-in-differences parallel trends assumption. We tested whether socioeconomic deprivation moderated any effect of the law change on total RTA rates by including an interaction term between the interventional group indicator and socioeconomic deprivation in our statistical models.

We separately fitted time-series seasonal autoregressive integrated moving average (SARIMA) models for off-trade and on-trade alcohol sales and for the interventional and control groups. SARIMA was considered the best model choice to account for the very strong seasonality in the alcohol consumption outcome. Logarithms of the outcome measures were used in our models to reduce the variability in the time series and to aid interpretation. We identified the form of the autocorrelation for the SARIMA errors from autocorrelation plots and partial autocorrelation plots. SARIMA was designed, for both interventional and control groups, in four different formats. Off-trade sales models controlled for off-trade sales of the other group, on-trade sales of the same group, and trend. Similarly, on-trade sales models controlled for on-trade sales of the other group, off-trade sales of the same group, and trend. We tested for residual correlation with correlograms to ensure that final models had a good fit with so-called white noise normally-distributed residuals (ie, residuals without serial correlation).

We used a statistical significance threshold of 0·05 throughout. We did all analyses with Stata/SE version 14.2 software. This study is registered with ISRCTN, number ISRCTN38602189.

### Role of the funding source

The funder of the study had no role in study design, data collection, data analysis, data interpretation, or writing of the report. The corresponding author had full access to all the data in the study and had final responsibility for the decision to submit for publication.

## Results

We assessed the weekly rate of RTAs, the rates of serious and fatal RTAs, single-vehicle night-time RTAs, and alcohol consumption in Scotland and England and Wales between Jan 1, 2013, and Dec 31, 2016, before and after the BAC limit came into effect in Scotland on Dec 5, 2014. The age, sex, and socioeconomic deprivation characteristics of drivers involved in the RTAs are shown in table 1. The distributions of these characteristics were very similar between drivers in RTAs in Scotland and those in England and Wales, which remained consistent in the sensitivity analysis that used the opposite demographic assignment rule (appendix). The mean number of drivers (and vehicles) per RTA was 1·72 (SD 0·73) in Scotland and 1·84 (0·71) in England and Wales. The corresponding number of casualties (ie, the number of people either killed or injured) per RTA was 1·29 (0·77) in Scotland and 1·33 (0·82) in England and Wales.

**Table 1:** Number of road traffic accidents by demographics of driver

	**Scotland (n=34 578)**	**England and Wales (n=527 068)**
**Sex**		
Male	27 075 (78·3%)	426 533 (80·9%)
Female	6938 (20·1%)	88 087 (16·7%)
Data missing	565 (1·6%)	12 448 (2·4%)
**Age group (years)**		
≤20	1716 (5·0%)	25 280 (4·8%)
21–25	2261 (6·5%)	38 457 (7·3%)
26–35	5326 (15·4%)	90 880 (17·2%)
36–45	6343 (18·3%)	102 268 (19·4%)
46–55	8180 (23·7%)	111 742 (21·2%)
56–65	5669 (16·4%)	71 698 (13·6%)
66–75	2798 (8·1%)	38 863 (7·4%)
>75	1792 (5·2%)	25 089 (4·8%)
Data missing	493 (1·4%)	22 791 (4·3%)
**Socioeconomic deprivation**		
1 (most deprived)	4687 (13·5%)	70 881 (13·4%)
2	4518 (13·1%)	69 663 (13·2%)
3	4118 (11·9%)	63 043 (12·0%)
4	3768 (10·9%)	57 301 (10·9%)
5	3343 (9·7%)	50 796 (9·6%)
6	3082 (8·9%)	45 228 (8·6%)
7	2647 (7·7%)	37 540 (7·1%)
8	2182 (6·3%)	31 417 (6·0%)
9	1735 (5·0%)	26 695 (5·1%)
10 (least deprived)	1402 (4·1%)	19 708 (3·7%)
Data missing	3096 (9·0%)	54 796 (10·4%)

Data are n (%). When an accident involved more than one driver, the demographic assignment was based on oldest age group, the most frequent sex, and most deprived socioeconomic deprivation group. An assessment based on the opposite demographic assignment is shown in the appendix.

**Figure 1:**
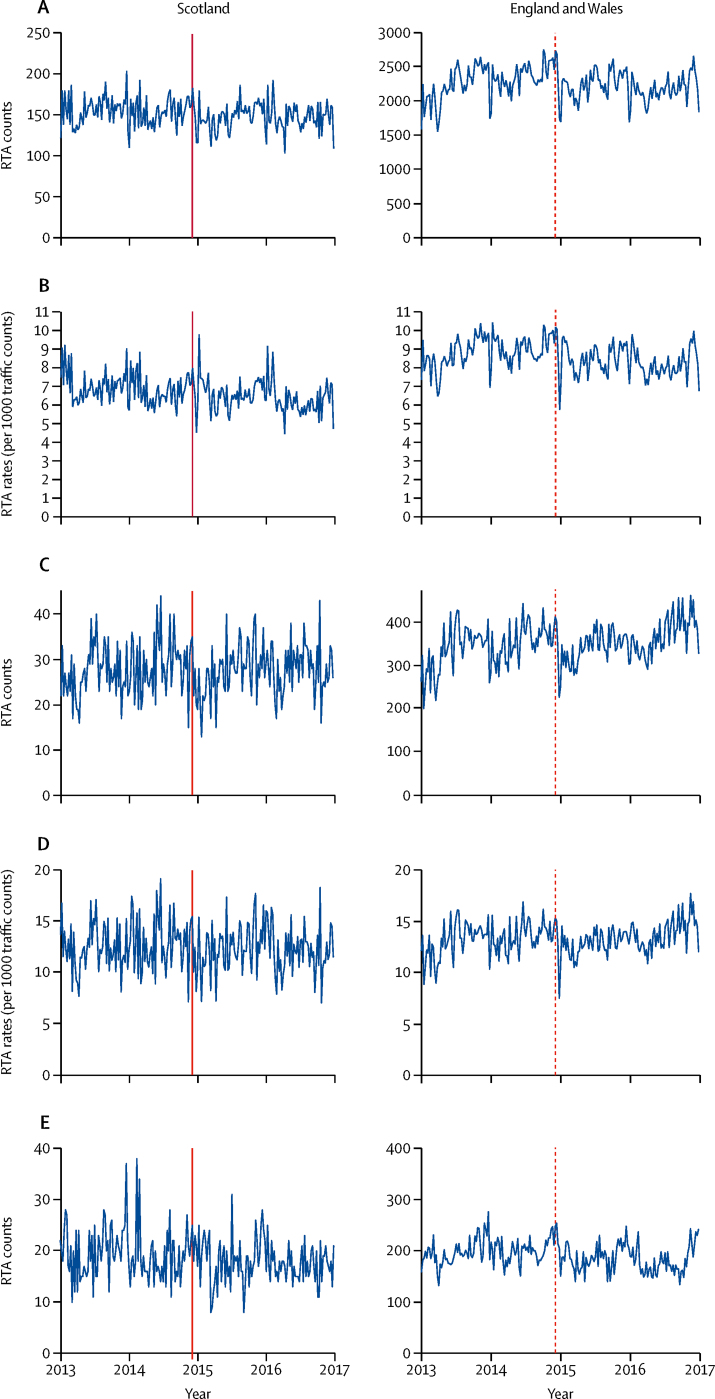
Weekly RTA counts (A), RTA rates (B), counts of serious or fatal RTAs (C), rates of serious or fatal RTAs (D), and single-vehicle night-time RTA counts (E), for Scotland and England and Wales between Jan 1, 2013, and Dec 31, 2016 The date of the change in legislation to reduce the blood alcohol concentration limit for drivers in Scotland is indicated by a solid vertical line and the equivalent date in the data for England and Wales is indicated by a dashed vertical line. RTA=road traffic accident.

Weekly RTA counts and rates relative to the date at which the reduction in the BAC limit for drivers was introduced are shown in figure 1. Between Jan 1, 2013, and Dec 31, 2016, the weekly RTA counts in Scotland were generally in the range of 100–200 RTAs, and the weekly counts in England and Wales were in the range 1500–2750 RTAs. Weekly RTA rates were higher in England and Wales than in Scotland during the study period; generally, there were 5–9 RTAs per 1000 traffic count in Scotland and 6–10 RTAs per 1000 traffic count in England and Wales. The weekly serious or fatal RTA counts in Scotland were typically in the range of 10–45 RTAs, and the weekly serious or fatal RTA counts in England and Wales were in the range of 200–450 RTAs. By contrast, during the 4 years of the study, the weekly rates of serious or fatal RTAs were comparable between groups. It is of note that, over the study period, fatal RTAs represented 1·9% of all RTAs in Scotland and 1·1% of all RTAs in England and Wales. It was not possible to calculate weekly single-vehicle night-time rates because the time of day was not recorded by the traffic counters, but we were able to evaluate weekly single-vehicle night-time counts. The weekly single-vehicle night-time RTA counts in Scotland were mostly in the range of 10–40 RTAs, and the weekly serious or fatal RTA counts in England and Wales were in the range of 130–275 RTAs.

We found that the reduction in BAC limit for drivers was not associated with a significant change in weekly RTA rates in Scotland, after adjustment for seasonality and underlying temporal trend (model c; rate ratio [RR] 1·01, 95% CI 0·94–1·08; p=0·77; table 2). Further, relative to England and Wales, where the reduction in BAC limit for drivers did not occur, we found no change in weekly RTA rates after this reduction in BAC limit for drivers in Scotland (model c; 1·07, 0·98–1·17; p=0·10). Further adjustment for the driver characteristics of age, sex, and socioeconomic deprivation produced similar results, showing a significant 7% increase in weekly RTA rates in Scotland relative to England and Wales (model d; 1·07, 1·02–1·13; p=0·007).

**Table 2:** Modelling results for RTA counts, RTA rates, and alcohol consumption

	**Scotland**		**England and Wales**		**Differences-in-differences (Scotland/England and Wales)**	
	Effect size (95% CI)	p value	Effect size (95% CI)	p value	Effect size (95% CI)	p value
**RTA counts**						
Model a	0·98 (0·91 to 1·04)	0·53	0·95 (0·90 to 1·00)	0·05	NA	NA
Model b	0·98 (0·93 to 1·03)	0·40	0·95 (0·93 to 0·96)	<0·0001	NA	NA
**RTA rates**						
Model c	1·01 (0·94 to 1·08)	0·77	0·94 (0·89 to 0·99)	0·02	1·07 (0·98 to 1·17)	0·10
Model d	1·00 (0·96 to 1·06)	0·73	0·94 (0·92 to 0·96)	<0·0001	1·07 (1·02 to 1·13)	0·007
**Serious or fatal counts of RTAs**						
Model e	0·90 (0·80 to 1·02)	0·10	0·90 (0·85 to 0·96)	0·001	NA	NA
Model f	0·90 (0·80 to 1·01)	0·08	0·90 (0·87 to 0·94)	<0·0001	NA	NA
**Serious or fatal rates of RTAs**						
Model g	0·93 (0·82 to 1·05)	0·24	0·89 (0·84 to 0·95)	0·0002	1·04 (0·90 to 1·19)	0·59
Model h	0·93 (0·83 to 1·04)	0·21	0·89 (0·87 to 0·92)	<0·0001	1·04 (0·92 to 1·17)	0·54
**Single-vehicle night-time RTA counts**						
Model i	0·99 (0·87 to 1·15)	0·99	0·93 (0·88 to 0·99)	0·03	NA	NA
Model j	0·99 (0·87 to 1·14)	0·99	0·93 (0·89 to 0·97)	0·002	NA	NA
**Per-capita alcohol sales**						
Model k (off-trade)	−0·003 (−0·017 to 0·011)	0·71	0·012 (−0·005 to 0·029)	0·18	NA	NA
Model l (on-trade)	−0·007 (−0·008 to −0·005)	<0·0001	0·007 (0·005 to 0·008)	<0·0001	NA	NA

Models a–j used negative binomial regression, and models k and l used seasonal autoregressive integrated moving averages. Models a, c, e, g, and i were adjusted for seasonality and underlying temporal trends. Models b, d, f, h, and j were adjusted for seasonality, underlying temporal trends, and driver characteristics (age, sex, and socioeconomic deprivation group). In Scotland, model k was adjusted for on-trade per-capita alcohol sales in Scotland and off-trade per-capita alcohol sales in England and Wales. In England and Wales, model k was adjusted for on-trade per-capita alcohol sales in England and Wales and off-trade per-capita alcohol sales in Scotland. In Scotland, model l was adjusted for off-trade per-capita alcohol sales in Scotland and on-trade per-capita alcohol sales in England and Wales. In England and Wales, model l was adjusted for off-trade per-capita alcohol sales in England and Wales and on-trade per-capita alcohol sales in Scotland. RTA=road traffic accident. NA=not applicable.

The differences-in-differences type estimate for the rates of serious or fatal RTAs indicated no significant difference in the intervention effect for Scotland relative to England and Wales both when adjusting for seasonality and underlying temporal trend (model g; RR 1·04, 95% CI 0·90–1·19; p=0·59; table 2), and when also adjusting for the driver characteristics of age, sex, and socioeconomic deprivation (model h; 1·04, 0·92–1·17; p=0·54). In a sensitivity analysis, we found similar results when modelling the rates of serious and fatal RTAs separately (appendix). For single-vehicle night-time RTA counts, the models showed no significant difference in Scotland (model j; 0·99, 0·87–1·15; p=0·99) but indicated a significant psuedo-intervention effect in England and Wales (model i; 0·93, 0·88–0·99; p=0·03) after the reduction in the BAC limit for drivers. Adjustment for age, sex, and socioeconomic deprivation did not have a significant effect on these results (table 2), and nor did changing the demographic assignment rule (appendix). Further, we observed similar null effects for Scotland when we modelled single-vehicle night-time relative to multi-vehicle day-time and multi-vehicle-alone outcomes (appendix). We found no evidence of effect modification by socioeconomic deprivation for total RTA rates (appendix).

**Figure 2:**
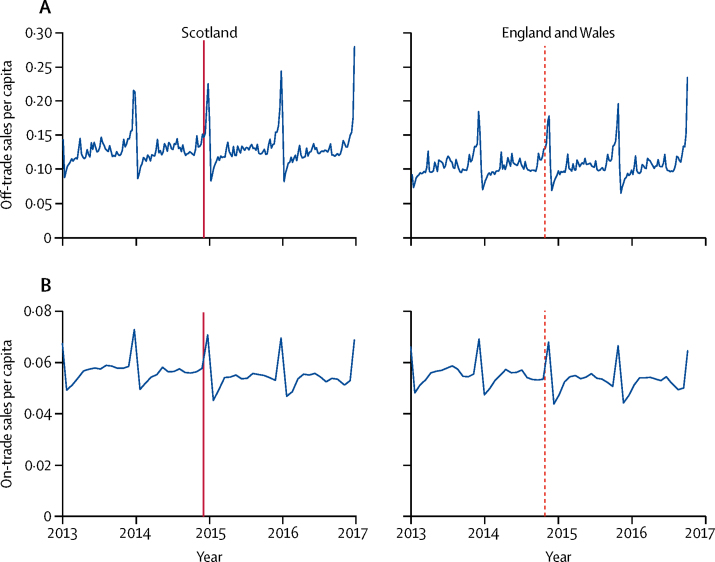
Weekly off-trade (A) and on-trade (B) per-capita alcohol sales in Scotland and England and Wales between Jan 1, 2013, and Dec 31, 2016 The date of the change in legislation to reduce the blood alcohol concentration limit for drivers in Scotland is indicated by a solid vertical line and the equivalent date in the data for England and Wales is indicated by a dashed vertical line.

The weekly off-trade and on-trade per capita alcohol sales during the study are shown in figure 2. We found strong seasonal patterning in alcohol sales, with large peaks at the end of the calendar year and smaller troughs at the start of the calendar year. The change in legislation in Scotland was associated with no significant change in per-capita off-trade sales (−0·3%, 95% CI −1·7 to 1·1; p=0·71) but a 0·7% decrease in per-capita on-trade sales (−0·7%, −0·8 to −0·5; p<0·0001). The corresponding results for the effect of the pseudo change in legislation in England and Wales indicated significant increases in per-capita on-trade sales (0·7%, 0·5 to 0·8; p<0·0001) but not in off-trade sales (1·2%, −0·5 to 2·9; p=0·18).

## Discussion

We found that lowering the BAC limit for drivers from 0·08 g/dL to 0·05 g/dL in Scotland did not decrease the rate of RTAs in the first 2 years after this legislation change. Our negative findings for total, serious or fatal, and single-vehicle night-time RTAs were unexpected given previous evidence that generally supports a reduction of RTAs after reducing the BAC limit for drivers. The 95% CIs of our results (table 2) do not include effect sizes of the magnitude that were reported by Henstridge and colleagues^7^ or Albalate.^12^ We found no evidence of an effect of this intervention on off-trade alcohol sales, and these sales account for a large proportion of alcohol consumption in Scotland (73% of total alcohol sales in 2017).^22^ We did observe a small reduction (of less than 1%) in on-trade alcohol sales, and further research is underway to explore perceptions of the effects of the change in the BAC limit for drivers from the perspectives of owners and managers of on-trade establishments.

Our study used a well designed natural experiment, with England and Wales providing a counterfactual for RTA and alcohol consumption trends in the absence of the BAC intervention. The same data sources were used for both Scotland and England and Wales, which helped to reduce measurement error. The distribution of demographic characteristics was very similar between the drivers in RTAs in Scotland and those in England and Wales, which further validates the appropriateness of the control group. With our large nationally representative data sets and with 2 years of weekly data points before and after the legislation change, these data had high statistical power, resulting in good precision around effect size estimates. The long follow-up means that it is unlikely that we have missed any lagged effect of the intervention.

Our study has limitations; first, we were unable to use alcohol-related RTAs as an outcome measure. BAC levels in drivers or riders involved in RTAs are often not available or are unreliable.^7^ For example, in the UK, only half of all drivers or riders involved in RTAs are breath tested by police^23^ and, for fatal RTAs, there are often long delays in attaining BAC from coroners' reports, and BAC naturally reduces with time. Moreover, in the STATS19 form, the BAC of the driver is not recorded; more crudely, whether the reading was in excess of the BAC limit is recorded. These data would present a methodological challenge since the limit changed in Scotland. Second, we have not adjusted for potential temporally confounding factors, such as weather and road quality. These factors would only be important if they were substantially different in the interventional and control groups, and we do not think this is likely. Further, we are not aware of any other concurrent interventions that were being used in Scotland and not in England and Wales, or vice versa. Third, we acknowledge that not all RTAs will become known to the police,^24^ and many casualties of RTAs who attend hospital will not be recorded in STATS19 forms. However, these omissions would only bias our results if they differed between the interventional groups, which is unlikely. Finally, the traffic flow denominators obtained from automatic traffic counters are a proxy for distance travelled by each person at risk of having an RTA, and there were data quality issues with the denominator that we used for the rates (which were addressed by multiple imputation); however, these traffic flow counts are superior to use of a population denominator because they more accurately reflect those at risk of RTAs. Moreover, although the location of the (approximately) 300 automatic traffic counters across the UK are placed to be broadly representative of the entire road network, they provide only a set of point estimates. Nevertheless, they provide good data on how traffic flows vary on a temporal basis and it is notable that the effect sizes we obtained from models using them closely match the results from modelling RTA counts (table 2).

The most plausible explanation of our finding of no effect of the reduced BAC limit for drivers on RTA outcome is that this limit was insufficiently enforced or publicised or both. A European Union strategy to support member states in reducing alcohol-related harm stated that a key to the success of drink-drive legislation after its introduction is the enforcement of frequent and systematic random breath testing, supported by public education, publicity, and awareness campaigns that involve all stakeholders.^25^ Further, previous research^9^ supports an association between increased enforcement and decreased RTAs. Random breath testing is recognised as the principal drink driving law enforcement strategy. Most of the decrease in alcohol-related traffic injuries and fatalities in Australia, for instance, has been attributed to the implementation of random breath tests.^26^, ^27^ There is evidence that enforcement of BAC limits for drivers has reduced in the UK, with English police force data^28^ showing 25% fewer breath tests in 2015 compared with 2011. In Scotland, the initial investment in public education and media campaigning at the time of the limit reduction in December, 2014 was not maintained in the subsequent years. Other explanations are, first, that the majority of drink driving RTAs (RTAs caused by drivers with a BAC in excess of the legal limit) might be caused by people who continue to ignore the law under the new legislation, or that people who previously used to drink-drive between the new and old limits have changed their behaviours but are responsible for only a small fraction of all RTAs. Second, it could be that larger effect sizes seen historically for BAC lowering interventions might be more difficult to achieve in an era of improved road safety and where drink driving is increasingly socially unacceptable. Finally, it could be that RTAs that are not related to alcohol have increased in Scotland during the study period, masking an intervention effect; however, given the modelling results, we think this explanation is unlikely. Further research exploring these and other possible explanations for the findings is needed.

Our findings indicate that the reduction in Scotland's BAC limit for drivers in December, 2014 did not have the intended effect of reducing RTAs. This limit reduction was associated with a reduction in on-trade alcohol sales by less than 1%, but there was no change in off-trade sales (which account for approximately three-quarters of total sales). This finding suggests that a reduction in BAC limit from 0·08 g/dL to 0·05 g/dL is not effective in reducing RTAs without being accompanied by other measures, such as enhanced enforcement. Our findings have important policy implications internationally as several countries and jurisdictions consider a similar reduction in BAC limit for drivers.
