# Supplementary material for: An evaluation of the effects of lowering blood alcohol concentration limits for drivers on the rates of road traffic accidents and alcohol consumption: a natural experiment
Source: Lancet. 2019 Jan 26;393(10169):321–9. doi: 10.1016/S0140-6736(18)32850-2 (PMC6346081; doi:10.1016/S0140-6736(18)32850-2)
Supplement: Supplementary appendix [file mmc1.pdf]

THE LANCET

Supplementary appendix

This appendix formed part of the original submission and has been peer reviewed. We post it as supplied by the authors.

Supplement to: Haghpanahan H, Lewsey J, Mackay DF, et al. An evaluation of the effects of lowering blood alcohol concentration limits for drivers on the rates of road traffic accidents and alcohol consumption: a natural experiment. *Lancet* 2018; published online Dec 12. http://dx.doi.org/10.1016/S0140-6736(18)32850-2.

Table S1: Number of road traffic accidents by demographics of driver

		Scotland (n = 34,578)	England & Wales (n = 527,068)
Sex	Male	9,039 (26·1)	101,420 (19·2)
	Female	24,974 (72·3)	413,200 (78·4)
	Missing	565 (1·6)	12,448 (2·4)
Age group (years)	< 20	4,653 (13·4)	75,155 (14·3)
	21–25	5,560 (16·1)	95,099 (18·0)
	26–35	9,129 (26·4)	142,168 (27·0)
	36–45	6,331 (18·3)	89,037 (16·9)
	46–55	4,847 (14)	59,370 (11·3)
	56–65	2,202 (6·4)	25,673 (4·9)
	66–75	876 (2·5)	11,122 (2·1)
	> 75	487 (1·4)	6,653 (1·3)
	Missing	493 (1·4)	22,791 (4·3)
Socio-economic deprivation	1 (Most deprived)	2,038 (6·0)	30,145 (5·7)
	2	2,476 (7·2)	38,228 (7·3)
	3	2,645 (7·6)	42,216 (8·0)
	4	3,024 (8·7)	45,461 (8·6)
	5	3,183 (9·2)	47,227 (9·0)
	6	3,515 (10·2)	49,942 (9·5)
	7	3,517 (10·2)	51,408 (9·7)
	8	3,663 (10·6)	53,754 (10·2)
	9	3,884 (11·2)	56,955 (10·8)
	10 (Least deprived)	3,628 (10·5)	56,936 (10·8)
	Missing	3,096 (8·9)	54,796 (10·4)

Data are n (%). An accident can involve more than one driver. Where this occurs, the demographic assignment was based on youngest age group, the least frequent sex, and highest (least deprived) socio-economic deprivation group.

Table S2: Modelling results for RTA counts, RTA rates, RTA serious/fatal counts, RTA serious/fatal rates and SVN RTA counts

**Models**	**Scotland**		**England & Wales**		**Difference-in-difference (Scotland / England & Wales)**	
	Effect Size (95% CI)	p-value	Effect Size (95% CI)	p-value	Effect Size (95% CI)	p-value
(a) RTA counts	0·98 (0·91, 1·04)	0·53	0·95 (0·90, 1·00)	0·05	NA	NA
(b) RTA counts	0·98 (0·93, 1·03)	0·42	0·95 (0·93, 0·98)	<0·001	NA	NA
(c) RTA rates	1·01 (0·94, 1·08)	0·77	0·94 (0·89, 0·99)	0·02	1·07 (0·98, 1·17)	0·10
(d) RTA rates	1·01 (0·96, 1·06)	0·72	0·94 (0·92, 0·97)	<0·001	1·07 (1·01, 1·13)	0·02
(e) RTA serious/fatal counts	0·90 (0·80, 1·02)	0·10	0·90 (0·85, 0·96)	<0·001	NA	NA
(f) RTA serious/fatal counts	0·91 (0·81, 1·02)	0·11	0·91 (0·88, 0·94)	<0·001	NA	NA
(g) RTA serious/fatal rates	0·93 (0·82, 1·05)	0·24	0·89 (0·84, 0·95)	<0·001	1·04 (0·90, 1·19)	0·59
(h) RTA serious/fatal rates	0·94 (0·84, 1·05)	0·28	0·90 (0·87, 0·93)	<0·001	1·04 (0·93, 1·18)	0·48

(i) SVN RTA counts	0·99 (0·87, 1·15)	0·99	0·93 (0·88, 0·99)	0·03	NA	NA
(j) SVN RTA counts	0·99 (0·87, 1·14)	0·99	0·93 (0·89, 0·97)	0·002	NA	NA

Negative binomial regression was employed for models a-j. Models a, c, e, g & i adjusted for seasonality, underlying temporal trend. Models b, d, f, h & j adjusted for seasonality, underlying temporal trend, age of driver, sex of driver and socio-economic deprivation group of driver. Demographic assignment was based on youngest age group, least frequent sex, and least deprived socio-economic deprivation group.

Table S3: Modelling results for SVN/MVD, MVD RTA count, serious RTA rates and fatal RTA rates

**Models**	**Scotland**		**England & Wales**		**Difference-in-difference (Scotland / England & Wales)**	
	Effect Size (95% CI)	p-value	Effect Size (95% CI)	p-value	Effect Size (95% CI)	p-value
(a) SVN/MVD	1·06 (0·76, 1·47)	0·75	0·98 (0·80, 1·20)	0·84	1·08 (0·73, 1·59)	0·70
(b) SVN/MVD	0·90 (0·72, 1·14)	0·41	0·98 (0·91, 1·05)	0·54	0·92 (0·72, 1·19)	0·55
(c) MVD RTA count	0·95 (0·95, 1·05)	0·31	0·94 (0·88, 1·00)	0·07	NA	NA
(d) MVD RTA count	0·97 (0·89, 1·05)	0·50	0·94 (0·92, 0·97)	<0·001	NA	NA
(e) serious RTA rates	0·94 (0·82, 1·06)	0·32	0·89 (0·84, 0·94)	<0·001	1·05 (0·91, 1·21)	0·46
(f) serious RTA rates	0·94 (0·83, 1·05)	0·29	0·89 (0·86, 0·92)	<0·001	1·05 (0·93, 1·19)	0·40
(g) fatal RTA rates	0·87 (0·62, 1·24)	0·45	0·99 (0·87, 1·20)	0·86	0·88 (0·61, 1·28)	0·51
(h) fatal RTA rates	0·87 (0·62, 1·23)	0·44	0·99 (0·88, 1·11)	0·85	0·88 (0·61, 1·27)	0·50

Gamma regression and negative binomial regression was employed for models a & b and c-h, respectively. Models a, c, e & g adjusted for seasonality, underlying temporal trend. Models b, d, f, & h adjusted for seasonality, underlying temporal trend, age of driver, sex of driver and socio-economic deprivation group of driver. Demographic assignment based on eldest age group, the most frequent sex, and lowest (most deprived) socio-economic deprivation group. In models a & b the covariates of age of driver, sex of driver and socio-economic deprivation group of driver had two levels each due to models with finer categorisation (see Table 1) not converging.

Table S4: Modelling results by level of socio-economic deprivation (interaction model)

Models		Scotland		England & Wales	
	Socio-economic deprivation (quintile)	Effect Size (95% CI)	p-value	Effect Size (95% CI)	p-value
RTA counts	1st	1·00 (0·92, 1·10)	0·93	0·96 (0·94, 0·99)	0·008
	2nd	1·00 (0·90, 1·10)	0·95	0·96 (0·93, 0·98)	0·004
	3rd	0·93 (0·83, 1·04)	0·19	0·94 (0·91, 0·97)	<0·001
	4th	1·01 (0·87, 1·15)	0·90	0·93 (0·90, 0·97)	0·001
	5th	0·92 (0·79, 1·08)	0·32	0·94 (0·90, 0·98)	0·01
RTA rates	1st	1·03 (0·95, 1·13)	0·44	0·95 (0·93, 0·98)	0·001
	2nd	1·03 (0·93, 1·13)	0·59	0·95 (0·92, 0·97)	<0·001
	3rd	0·96 (0·86, 1·07)	0·44	0·93 (0·90, 0·96)	<0·001
	4th	1·04 (0·91, 1·18)	0·56	0·93 (0·89, 0·96)	<0·001
	5th	0·95 (0·81, 1·11)	0·54	0·93 (0·89, 0·97)	0·003

Demographic assignment based on eldest age group, the most frequent sex, and lowest (most deprived) socio-economic deprivation group.

Table S5: Modelling results by level of socio-economic deprivation (interaction model)

Models		Scotland		England & Wales	
	Socio-economic deprivation (quintile)	Effect Size (95% CI)	p-value	Effect Size (95% CI)	p-value
RTA counts	1st	1·03 (0·91, 1·18)	0·61	1·00 (0·95, 1·04)	0·92
	2nd	0·98 (0·87, 1·10)	0·77	0·96 (0·92, 1·00)	0·03
	3rd	0·97 (0·87, 1·08)	0·60	0·95 (0·92, 1·00)	0·01
	4th	1·00 (0·90, 1·11)	0·96	0·95 (0·91, 0·98)	0·006
	5th	0·93 (0·87, 1·03)	0·14	0·93 (0·90, 0·96)	<0·001
RTA rates	1st	1·06 (0·93, 1·22)	0·33	0·99 (0·94, 1·03)	0·59
	2nd	1·01 (0·90, 1·14)	0·84	0·95 (0·91, 0·98)	0·008
	3rd	1·00 (0·90, 1·11)	0·98	0·94 (0·91, 0·98)	0·002
	4th	1·03 (0·93, 1·15)	0·53	0·94 (0·90, 0·97)	0·001

5th	0·95 (0·86, 1·06)	0·37	0·92 (0·89, 0·95)	<0·001

Demographic assignment was based on youngest age group, the least frequent sex, and highest (least deprived) socio-economic deprivation group.
